# Tobacco use trends in South Korea, 2013–2023: Persistent disparities and emerging challenges in a repeated cross-sectional study

**DOI:** 10.18332/tid/215655

**Published:** 2026-02-06

**Authors:** Boram Lee, Mijeong Kwon, Ah-Hyun Park, Hyekyeong Kim

**Affiliations:** 1 Department of Health Convergence, Ewha Womans University, Seoul, Republic of Korea; 2 Seoul Tobacco Control Center, Seoul, Republic of Korea

**Keywords:** tobacco use prevalence, tobacco disparities, trends in tobacco use, Korea

## Abstract

**INTRODUCTION:**

The emerging non-combustible tobacco products have complicated the tobacco landscape in Korea. This study aimed to assess subgroup trends in conventional cigarette (CC), electronic cigarette (EC), heated tobacco product (HTP), and poly-tobacco use in South Korea from 2013 to 2023.

**METHODS:**

We analyzed secondary data from the 2013–2023 Korea National Health and Nutrition Examination Survey, a nationally-representative cross-sectional data of adults (aged ≥19 years) (n=62935). Joinpoint regressions were used to estimate average annual percent changes (AAPCs) in tobacco use, stratified by sociodemographic and health-related characteristics. All measures were based on self-reports.

**RESULTS:**

Among men, the prevalence of CC smoking declined from 42.1% in 2013 to 32.2% in 2023 (AAPC= -3.4; 95% CI: -4.6 – -2.3), with small declines among those with a lower income, less-educated, manual workers, and those with multiple risk behaviors, and severe mental illness. EC use increased modestly overall, with a significant rise among in men aged 25–39 years; HTP use showed a slight overall decline. Poly-tobacco use increased, particularly among young adults (aged 19–24 years) and middle-aged adults (aged 40–64 years) the lowest-income group, manual workers, and those with multiple risk behaviors. Among women, overall prevalence of CC, EC, and HTP use remained below 7%, but prevalence rose among young women aged 19–24 years (CC: 9.6% to 16.1%; EC: 0.7% to 5.6%; HTP: 3.1% to 5.8%), although the corresponding AAPCs were not statistically significant (CC: 4.6; 95% CI: -1.9–12.4; EC: 15.1; 95% CI: -0.1–39.7; HTP: 26.3; 95% CI: -21.4–125.0).

**CONCLUSIONS:**

Korea's progress in reducing CC smoking has not extended to vulnerable populations, and rising EC and poly-tobacco use, particularly among young adults, present new challenges. These findings underscore the need for tailored cessation interventions for vulnerable populations and for ongoing efforts to tackle the emerging use of novel tobacco products.

## INTRODUCTION

Tobacco use is a global public health threat, accounting for more than 7.7 million annual deaths worldwide^1^. Although smoking prevalence has largely decreased in most high-income countries over the past three decades, some countries still report high smoking rates, particularly among men^1,2^. South Korea ranked fourth in male smoking prevalence among high-income countries, with rates comparable to those in many low- and middle-income countries^1^. Since ratifying the World Health Organization’s Framework Convention on Tobacco Control (FCTC) in 2005, South Korea has implemented a series of tobacco control policies, including cigarette tax increases, pictorial health warnings on tobacco products, nationwide smoking cessation services, and smoke-free policies in public places^3^. These efforts have contributed to a decline in adult conventional cigarette smoking prevalence from 28.8% (male 51.7%, female 5.7%) in 2005 to 17.7% (male 30.0%, female 5.0%) in 2022. However, the downward trend stalled, and, indeed, the prevalence rebounded to 19.6% (male 32.4%, female 6.3%) in 2023^4^, which may reflect a waning impact of prior tobacco control measures and slower progress in complete smoking cessation, partly due to the expanding availability of novel tobacco products^3^.

The growing availability of electronic cigarettes (ECs) and heated tobacco products (HTPs) increases combined use of conventional cigarettes (CCs) with EC or HTPs (hereafter, poly-tobacco use) and appears to impede further declines in CC smoking prevalence^5,6^. The rapid proliferation of novel tobacco products, such as ECs and HTPs has complicated the tobacco use landscape in Korea. Following the introduction of HTPs in Korea in 2017, sales of ECs and HTPs have increased by 8.3 times, from 78.7 million to 655.9 million packs^7^. These shifts in the tobacco market underscore the need for a robust understanding of how tobacco use patterns have evolved over time in South Korea, and the extent to which these patterns have been equally distributed across the population.

Prior studies on trend analyses have been limited in capturing recent use across different tobacco products (CCs, ECs, HTPs, and poly-tobacco products) and in evaluating specific trends within subgroups based on sociodemographic and health-related characteristics, such as mental illness and clustering of unhealthy behaviors^3,8^. Specifically, most previous studies did not examine poly-tobacco use or subgroup analyses were largely restricted to sex and age^3,8^. Tobacco use is more prevalent among individuals with mental illness, as it is often used as a means of coping with psychological distress^9^, and tobacco use tends to cluster with other health risk behaviors^10^. Consequently, individuals with mental illness and multiple unhealthy behaviors represent critical subgroups for monitoring tobacco use trends; however, long-term trends in tobacco use among these subgroups has been largely overlooked in prior studies^10,11^. In this study, we aimed to examine trends in tobacco use over the past ten years (2013–2023) in South Korea, focusing on CCs, ECs, HTPs, and dual or triple use of these products, stratified by key sociodemographic and health-related characteristics. The findings of this study may contribute to identifying vulnerable subgroups with higher or increasing tobacco use in South Korea and to improving the understanding of tobacco use patterns in Asian countries where male smoking remains high and novel tobacco products have been increasingly available on the tobacco market.

## METHODS

### Data and sample

**Figure 1:**
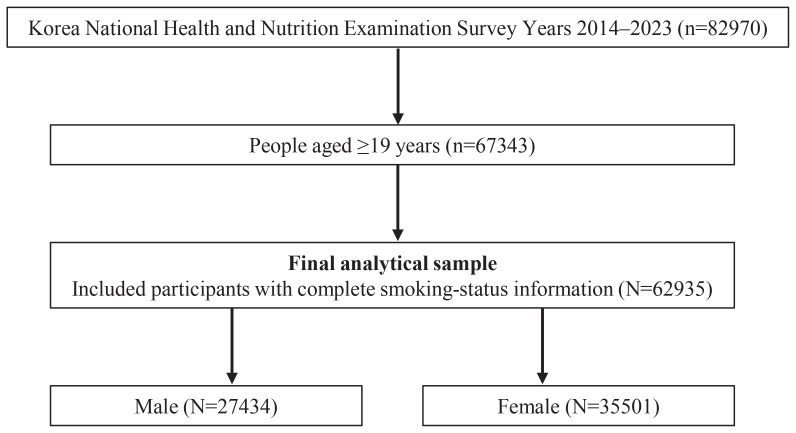
Flowchart of participant selection for the final analytic sample

This study is a secondary data analysis using data from the 2013–2023 Korea National Health and Nutrition Examination Survey (KNHANES), a nationally representative ongoing annual cross-sectional survey, conducted by the Korea Disease Control and Prevention Agency^4^. Since 1998, KNHANES has collected information on health behaviors and health status through standardized health interviews and examinations by trained interviewers^4^. The KNHANES employs a stratified multistage clustered probability sampling design to select nationally representative households, with the target population of non-institutionalized Korean citizens. Details can be found elsewhere^12^. Of the initial participants (n=82970), we excluded those younger than 19 years of age (15627 respondents) and those with missingness on tobacco use (4408 respondents), reducing the analytic sample of Korean adults (≥19 years) to 62935 (Figure 1). This study was deemed exempt by the Institutional Review Board at Ewha Womans University from ethical approval.

### Measures

*Tobacco use*

Current use of conventional cigarettes (CCs), electronic cigarettes (ECs), and heated tobacco products (HTPs) was assessed using self-reported responses collected by trained interviewers. Individuals who reported having smoked at least 100 cigarettes in their lifetime and currently smoked CCs either every day or some days were classified as current CC smokers. Those who answered ‘yes’ to the questions ‘do you currently use e-cigarettes?’ and ‘do you currently use heated tobacco products?’ were classified as current EC users and current HTP users, respectively. As HTP uses have been assessed since 2019 in KNHANES, related analyses were limited to 2019 onward. Poly-tobacco use in the present study refers to the concurrent use of more than one tobacco product (dual and triple use of CCs, ECs, and HTPs).

*Sociodemographic and health-related characteristics*

We estimated annual prevalence of tobacco use by sex (female, male), age groups (19–24, 25–39, 40–64, and ≥65 years), household income quartile, education level (high school or less, college or higher), occupational groups (non-manual workers, manual workers, other), number of health risk behaviors (none, one, two), and mental illness (low, moderate, severe). Non-manual workers included professionals, managers, office workers; manual workers included service and sales workers, agricultural workers, craft workers, machine operators, and elementary occupations; and other included students, homemakers, and the unemployed^13^. The number of health risk behaviors were calculated based on the presence of excessive drinking (drinking at least twice a month with ≥7 drinks for men or ≥5 for women on one occasion) and physical inactivity (<150 minutes of aerobic physical activity per week), ranging from 0 (none) to 2 (both)^13^. Mental illness was classified based on two indicators: depressive symptoms, defined as sadness or hopelessness lasting at least two weeks in the past year, and psychological distress, defined as experiencing ‘very much’ or ‘much’ distress in daily life^13,14^. Mental illness was categorized as low when neither depressive symptoms nor psychological distress were reported, moderate when one condition was reported, and severe when both were reported^14^. As assessment of mental illness was conducted only in even-numbered years, analyses involving mental illness were restricted to those years^13^.

### Statistical analysis

The annual prevalence of CC, EC, HTP, and poly-tobacco use was estimated by subgroups based on sociodemographic and health-related characteristics. Trends in the annual prevalence of tobacco use from 2013 to 2023 were quantified for each subgroup using the joinpoint regressions with average annual percent changes (AAPCs) and 95% confidence intervals (CIs). The final model was selected by comparing Bayesian information criterion (BIC) values between similar models and more complex models^15^. Given the substantial heterogeneity in tobacco use between Korean men and women, all subgroup analyses were conducted separately for each sex. All analyses were weighted applying the sampling weights provided by KNHANES to reflect the complex survey design^13^, and conducted using SPSS Statistics version 27.0 (IBM Corp., Armonk, NY) and the National Cancer Institute Joinpoint Regression Program, version 5.2.0. A two-sided p<0.05 was considered statistically significant.

## RESULTS

**Figure 2:**
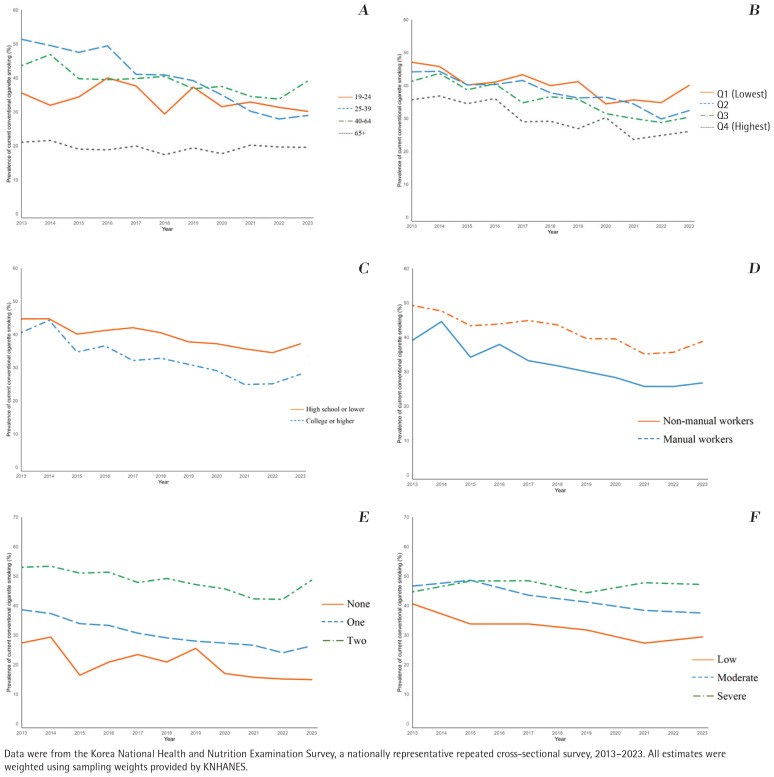
Trends in smoking prevalence among adult males by (A) age, (B) income, (C) education, (D) occupation, (E) the number of health risk behaviors, and (F) mental illness in South Korea, 2013-2023 (N=27434)

Of the 62935 adults considered in the present study, 50.4% were female, 9.4% were aged 19–24 years, 24.8% were in the lowest income quartile, 43.8% had an undergraduate degree, 36.1% were manual workers, 12.5% engaged in both excessive drinking and physical inactivity, and 7.3% reported severe mental illness (Supplementary file Table 1). Among Korean men, the prevalence of CC smoking declined by 9.9 percentage point (pp) from 42.1% in 2013 to 32.2% in 2023 (AAPC= -3.4; 95% CI: -4.6 – -2.3), with significant reductions in those aged 25–39 years (51.4% to 29.0%; AAPC: -6.0; 95% CI: -8.5 – -4.6) and 40–64 years (43.6% to 39.1%; AAPC= -2.2; 95% CI: -4.0 – -0.4) but not in young adults (aged 19–24 years) and older adults (aged ≥65 years) (Table 1 and Figure 2).

**Table 1:** Prevalence and trends of tobacco product use among male adults in South Korea, 2013-2023 (N=27434)

	*Traditional cigarette smoking*				*E-cigarette use*				*Heated tobacco product use*				*Poly-tobacco product use*			
	*2013*	*2023*	*Trend 2013–2023*		*2013*	*2023*	*Trend 2013–2023*		*2019*	*2023*	*Trend 2019–2023*		*2013*	*2023*	*Trend 2013–2023*	
	*%*	*%*	*AAPC*	*95% CI*	*%*	*%*	*AAPC*	*95% CI*	*%*	*%*	*AAPC*	*95% CI*	*%*	*%*	*AAPC*	*95% CI*
**Male**	42.1	32.2	-3.4***	-4.6 – -2.3	2.0	5.3	1.6	-3.8–7.8	8.8	7.7	-2.4	-10.5–5.9	1.8	6.9	9.10	-1.1–22.8
**Age** (years)																
19–24	35.6	30.2	-1.4	-3.4–0.6	0.8	5.9	16.3	-11.7–50.2	9.8	13.9	11.0	-7.5–33.1	0.8	12.5	8.5*	0.5–19.5
25–39	51.4	29.0	-6.0***	-8.5 – -4.6	2.6	12.8	6.5*	1.2–13.4	14.7	11.3	-6.7	-14.8–0.9	2.5	11.9	8.7	-0.9–23.1
40–64	43.6	39.1	-2.2*	-4.0 – -0.4	2.2	3.1	-1.5	-8.7–6.8	8.0	7.3	-0.2	-10.6–11.8	1.9	5.9	9.0***	2.7–17.9
≥65	21.1	19.7	-0.6	-2.6–1.7	1.1	0.1	-8.9	-23.9–4.2	0.4	0.9	13.2	-13.7–53.7	0.0	0.2	-4.7	-20.5–10.6
**Income level**																
Q1 (Lowest)	47.1	40.0	-2.3*	-4.5 – -0.2	2.7	4.8	2.4	-4.5–10.6	8.3	7.0	-3.9	-30.1–31.8	2.5	6.7	9.7*	1.2–21.1
Q2	44.1	32.4	-3.5***	-4.4 – -2.6	1.1	7.0	1.7	-7.0–11.8	7.9	8.5	5.9	-14.3–33.1	1.1	8.1	7.6	-4.8–24.1
Q3	41.3	30.4	-3.9***	-5.4 – -2.4	1.8	4.6	1.4	-5.8–10.2	10.4	7.2	-9.8*	-19.8 – -0.6	1.6	7.3	6.9	-5.3–24.3
Q4 (Highest)	35.7	26.0	-4.3***	-6.7 – -2.2	2.3	4.5	5.4	-1.1–13.3	8.8	7.9	-2.6	-11.9–5.9	2.1	5.4	8.2	-0.6–20.0
**Education level**																
≤ High school	44.7	37.2	-2.3***	-3.5 – -1.3	2.5	4.4	-0.2	-10.1–10.2	6.2	4.5	-6.7***	-11.1 – -2.6	2.2	5.8	5.6^#^	-0.3–12.8
≥ College	40.6	28.0	-5.0***	-6.6 – -3.5	1.6	6.0	4.2	-2.3–12.5	11.5	9.5	-4.2	-14.4–6.0	1.5	7.0	9.3^#^	-0.5–25.0
**Occupational group**																
Non-manual	39.1	26.7	-5.4***	-7.2 – -3.8	2.0	5.9	1.3	-7.4–11.7	12.4	10.1	-2.7	-18.0–13.9	1.8	7.5	7.2	-1.6–20.4
Manual	49.2	38.8	-2.9***	-4.4 – -1.6	2.1	5.4	2.5	-4.4–10.3	8.3	8.0	-0.4	-16.9–18.1	1.9	8.6	10.1*	1.9–21.6
Other	32.4	26.0	-2.0*	-3.7 – -0.3	1.8	3.8	2.5	-11.1–19.5	5.7	3.8	-10.6	-26.2–5.7	1.8	4.2	7.0	-6.0–26.0
**Number of risk behaviors**																
None	29.7	21.3	-4.2***	-6.2 – -2.3	1.0	3.9	4.1	-3.5–13.1	6.1	4.4	-8.9	-19.89–1.82	1.0	4.7	9.50	0.0–23.7
One	47.4	31.3	-4.5***	-6.1 – -3.0	2.3	5.6	2.5	-3.8–10.1	9.8	7.4	-4.9	-19.92–10.67	2.2	6.7	8.24	-1.8–22.9
Two	51.9	48.4	-2.0*	-3.5 – -0.4	3.1	6.2	-0.8	-6.7–6.1	10.1	13.3	8.2	-2.58–20.48	2.4	12.0	9.78*	0.8–22.4
**Mental illness**																
Low	40.6	29.4	-3.3*	-6.5 – -0.1	1.9	4.2	3.8	-2.8–11.6	7.7	6.4	-5.5	-17.05–6.02	1.8	5.6	9.09	-4.3–27.3
Moderate	46.6	37.5	-2.7***	-3.7 – -1.7	2.0	7.6	1.8	-13.1–19.8	11.4	9.6	-4.1***	-4.46 – -3.83	2.0	9.3	6.11	-18.0–42.7
Severe	44.7	47.2	0.0	-1.3–1.4	4.2	10.1	-5.3	-26.1–15.2	10.7	16.2	12.8	-15.54–59.85	2.0	15.1	0.65	-27.8–37.9

Data were from the Korea National Health and Nutrition Examination Survey (KNHANES), a nationally representative repeated cross-sectional survey, 2013–2023. All estimates were weighted using sampling weights provided by KNHANES. AAPC: average annual percent change.

# p<0.1,

* p<0.05,

** p<0.01,

*** p<0.001.

Throughout the study period, the CC prevalence remained consistently higher among those with a lower income, less-educated, and manual workers. The decline over the past decade was also smaller in those with the lowest level of income (47.1% to 40.0%; AAPC= -2.3, 95% CI: -4.5 – -0.2), lower level of education (44.7% to 37.2%; AAPC= -2.3, 95% CI: -3.5 – -1.3), and manual workers (49.2% to 38.8%; AAPC= -2.9; 95% CI: -4.4 – -1.6), relative to their counterparts. In addition, the reduced trends were smaller among men engaging in both excessive drinking and physical inactivity (51.9% to 48.4%; AAPC= -2.0; 95% CI: -3.5 – -0.4) than those without health risk behaviors (29.7% to 21.3%; AAPC= -4.2; 95% CI: -6.2 – -2.3). Notably, the CC prevalence remained unchanged over the decade among those with severe mental illness (44.7% to 47.2%; AAPC=0.0; 95% CI: -1.3–1.4).

EC use increased by 3.3 pp, from 2.0% in 2013 to 5.3% in 2023 among Korean men, with particularly increases in those aged 25–39 years (2.6% to 12.8%; AAPC=6.5; 95% CI: 1.2–13.4). HTP uses tended to be relatively unchanged from 2019 (8.8%) to 2023 (7.7%) with an AAPC of -2.4 (95% CI: -10.5–5.9), with a significant reduction in those with upper middle income (10.4% to 7.2%; AAPC= -9.8; 95% CI: -19.8 – -0.6), lower level of education (6.2% to 4.5%; AAPC= -6.7; 95% CI: -11.1 – -2.6), and moderate mental illness (11.4% to 9.6%; AAPC= -4.1; 95% CI: -4.5 – -3.8) (Table 1 and Supplementary file Figure 1).

**Figure 3:**
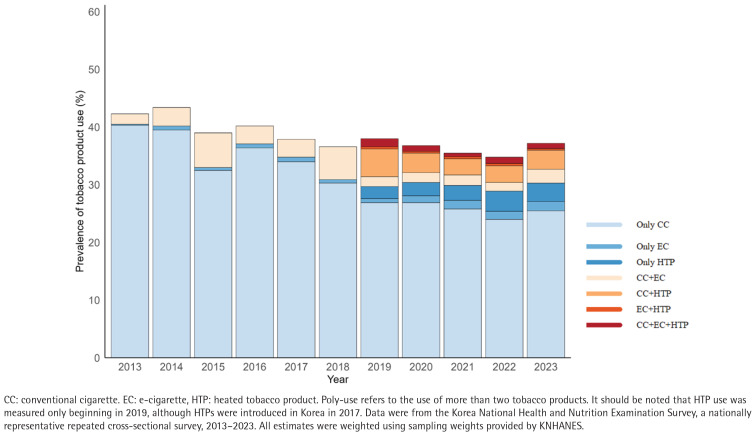
Prevalence of single- and poly-tobacco product use among adult men using nationally representative repeated cross-sectional surveys in South Korea, 2013–2023 (N=27434)

Figure 3 highlights the diversification of tobacco product use patterns among Korean men over the past ten years (2013–2023). Following availability of HTPs in 2019, while the proportion of men exclusively using CC decreased, the proportion of poly-tobacco use appeared to offset the reduced exclusive CC use. Trends in poly-tobacco use by subgroups among Korean men are presented in Table 1. Alarming increases in poly-tobacco use were most pronounced in young males aged 19–24 years (0.8% to 12.5%; AAPC=8.5; 95% CI: 0.5–19.5), adults aged 40–64 years (1.9% to 5.9%, AAPC=9.0; 95% CI: 2.7–17.9), low-income groups (2.5% to 6.7%, AAPC=9.7; 95% CI: 1.2–21.1), manual workers (1.9% to 8.6%, AAPC=10.1; 95% CI: 1.9–21.6), and those with multiple health risk behaviors (2.4% to 12.0%, AAPC=9.8; 95% CI: 0.8–22.4) (Table 1 and Supplementary file Figure 2).

For women, overall CC prevalence remained relatively unchanged from 2013 (6.8%) to 2023 (5.3%), but substantial differences were observed across age groups (Table 2). Over these ten years (2013–2023), CC prevalence significantly decreased from 4.2% to 2.1% (2.1 pp) among older women aged ≥65 years (AAPC= -6.8; 95% CI: -11.9 – -1.4), whereas the prevalence rose from 9.6% to 16.1% (6.5 pp) among young women aged 19–24 years (AAPC=4.6; 95% CI: -1.9–12.4). Although this upward trend among young women was not statistically significant, the magnitude of the increase in prevalence was noteworthy. Similarly, although the mean prevalence of EC (0.3% to 1.3%) and HTP use (1.5% to 2.1%) remained low among women overall, both appeared to increase among young women. EC use rose among women aged 19–24 years (0.7% to 5.6%, AAPC=15.1; 95% CI: -0.1–39.7) and 25–39 years (0.4% to 3.0%, AAPC=12.1; 95% CI: -0.4–28.0), although these trends did not reach statistical significance (p=0.052 and p=0.056, respectively). HTP use increased in women aged 25–39 years (3.1% to 4.1%, AAPC: 6.0; 95% CI: 0.2–11.6). Notably, female manual workers showed significant increases in both EC (0.6% to 2.2%) and HTP (1.7% to 3.8%) uses at AAPC of 12.4 (95% CI: 5.5–22.8) and 30.91 (95% CI: 7.6–71.9), respectively. Trends of poly-tobacco use and subgroup analyses by number of risk behaviors and mental illness could not be conducted for females due to their small sample size.

**Table 2:** Prevalence and trends of tobacco product use among female adults in South Korea, 2013–2023 (N=35501)

	*Traditional cigarette smoking*				*E-cigarette use*				*Heated tobacco product use*			
	*2013*	*2023*	*Trend 2013–2023*		*2013*	*2023*	*Trend 2013–2023*		*2019*	*2023*	*Trend 2019–2023*	
	*%*	*%*	*AAPC*	*95% CI*	*%*	*%*	*AAPC*	*95% CI*	*%*	*%*	*AAPC*	*95% CI*
**Female**	6.8	5.3	-1.5	-4.6–1.5	0.3	1.3	9.5	-1.4–23.5	1.5	2.1	12.4	-8.8–38.3
**Age**												
19–24	9.6	16.1	4.6	-1.9–12.4	0.7	5.6	15.1^#^	-0.1–39.7	3.1	5.8	26.3	-21.4–125.0
25–39	10.0	7.3	-0.6	-5.4–4.2	0.4	3.0	12.2^#^	-0.4–28.0	3.1	4.1	6.0*	0.3–11.6
40–64	5.3	4.2	-1.3	-5.3–2.6	0.4	0.4	-4.0	-22.6–12.9	1.0	1.5	20.2	-2.4–56.6
≥65	4.2	2.1	-6.8**	-11.9 – -1.4	0.1	0.1	-0.7	-8.3–13.2	0.0	0.0	-6.7	-23.1–12.9
**Income level**												
Q1 (Lowest)	10.0	9.1	-1.4	-4.9–2.3	0.3	0.9	8.9	-1.0–23.6	3.1	4.5	13.9	-0.7–35.3
Q2	7.2	5.1	-1.1	-7.6–5.1	0.8	1.9	6.7	-1.5–16.8	1.7	3.5	7.0	-6.5–20.7
Q3	5.1	4.7	-0.1	-2.8–3.0	0.1	1.2	6.4	-15.0–40.1	0.6	0.3	6.4	-23.8–50.0
Q4 (Highest)	5.0	2.5	-3.8	-10.9–3.5	0.0	1.1	14.5	-15.3–58.7	0.0	0.0	16.8	-0.8–41.0
**Education level**												
≤High school	7.7	5.5	-1.4	-4.9–2.2	0.4	0.8	4.4	-5.3–14.8	1.4	1.7	11.8	-14.0–46.7
≥College	3.8	3.0	-1.1	-4.3–2.2	0.2	1.2	10.0	-3.4–28.0	1.5	1.8	4.1	-5.5–15.4
**Occupational group**												
Non-manual	5.3	3.9	-1.9	-6.5–2.8	0.2	1.4	6.2	-11.3–31.3	2.1	1.6	-0.7	-25.2–32.1
Manual	9.9	7.4	-2.2	-5.1–1.2	0.6	2.2	12.4***	5.5–22.8	1.7	3.8	30.9***	7.6–71.9
Other	5.6	4.5	-1.7	-6.3–2.8	0.2	0.7	7.3	-13.1–33.0	1.2	0.9	-8.8	-20.5–1.8

Data were from the Korea National Health and Nutrition Examination Survey (KNHANES), a nationally representative repeated cross-sectional survey, 2013–2023. All estimates were weighted using sampling weights provided by KNHANES. AAPC: average annual percent change.

# p<0.1.

* p<0.05.

** p<0.01.

*** p<0.001.

## DISCUSSION

This study assessed ten-year trends in the use of CC, EC, HTPs, and poly-tobacco products across subgroups by sociodemographic and health-related characteristics in Korea, providing insights to help understand vulnerable groups with higher or increasing tobacco use in Korea. While overall tobacco use declined among men but remained unchanged among women over the ten-year period 2013–2023, trends varied across tobacco product types and subgroups. Among men, who account for 86% of Korean tobacco users, CC smoking substantially decreased; however, smoking became concentrated among socioeconomically disadvantaged groups (i.e. those with low income, lower level of education, or manual occupations), young adults (aged 19–24 years), and those with serious mental illness or other unhealthy risk behaviors.

The findings of persistent or even widened socioeconomic disparities in CC smoking underscore the need for stronger policies aimed at reducing tobacco use among socioeconomically disadvantaged populations in Korea. These findings are consistent with data from other developed countries (e.g. the United States), showing a higher tobacco burden among disadvantaged groups^16,17^, and contrast with data from some countries (e.g. Mexico) where tobacco use is more prevalent among affluent groups^18^. This discrepancy may reflect differences in strength of tobacco control policies, tobacco affordability, and perceptions of the harmfulness of tobacco use^18^. Our findings imply that the Korean government’s previous efforts to mitigate tobacco disparities were less effective than anticipated. Indeed, previous studies showed that the prior cigarette tax increase implemented in 2015, which raised CC prices by 80%, increased quit attempts among low-income smokers but did not lead to long-term abstinence^19^. Likewise, the regional tobacco control centers, established to improve access to tobacco cessation counseling for vulnerable populations through visiting (‘outreach’) services in Korea, appear to have limitations in reaching the vulnerable high-risk populations, partly due to limited resources and pressure to achieve high cessation success rates^20^.

The persistently high prevalence of CC use among individuals with severe mental illness is particularly concerning in Korea, which had the highest suicide rate among the 38 member countries in the Organization for Economic Cooperation and Development (OECD)^21^. Although elevated tobacco use in this group is commonly observed in other countries^17^, the lack of a decline in CC prevalence in Korea over a ten-year period, despite substantial reductions in other populations, is of particular concern. This stagnation underscores the urgent need to incorporate healthy stress coping strategies into cessation interventions as behavioral substitutes (‘counterconditioning’ in Transtheoretical model)^22^ and to raise public awareness that tobacco use exacerbates mental illness in the long-term^23^. In addition, the higher smoking prevalence and slower rate of decline among individuals with multiple health risk behaviors is noteworthy. As health risk behaviors, such as tobacco use, drinking alcohol, and physical inactivity tend to cluster and interact, addressing them in isolation may reduce cessation success^24^. Comprehensive interventions that concurrently target two or more risk behaviors (e.g. tobacco cessation with structured physical activity programs) should be considered, particularly for populations with high-risk profiles^10^.

Our findings demonstrate shifts in tobacco use landscape in Korea, with a decline in exclusive CC use and increases in EC and poly-tobacco use. The increases in EC use among young adults are concerning because they may indicate tobacco industry has expanded its user base in Korea^25^, making future reductions in tobacco prevalence more difficult to achieve. Prior evidence shows that young adults were strongly attracted to flavors in EC^26^, which lower barriers to initiation^27^ and reduce intention to quit^28^. A qualitative study of Korean young women found that flavored tobacco products reshaped their perception of tobacco use, from smoking to consuming products associated with taste and sensory satisfaction^27^. In addition, our results indicate that increases in poly-tobacco use were pronounced in young adults aged 19–24 years, middle-aged adults aged 40–64 years, low-income groups, manual workers, and those with multiple health risk behaviors among Korean men. Previous studies^29,30^ have shown that the main reasons for dual use are to reduce the perceived harm of tobacco use and avoid its smell; however, dual users are more likely than single-tobacco users to experience higher nicotine dependence and toxicant exposure^31-33^, lower intention to quit smoking^34^, and lower rates of successful abstinence^35^. These findings suggest that increased use of ECs and poly-tobacco products in vulnerable populations constitutes an important public health concern in Korea.

### Limitations

This study has several limitations. First, the small sample size of female smokers, reflecting the low prevalence of tobacco use among Korean women, may have reduced statistical power to detect subgroup-specific trends. It also precluded estimation of poly-tobacco trends and subgroup trends by number of health risk behaviors and severity of mental illness. Second, the timing of HTP introduction did not align with the survey period. Although HTPs were introduced in 2017, survey only measured HTP use from 2019^13^, limiting the ability to capture changes in tobacco use during the earliest adoption period. For example, the rapid decrease in combined use of CCs and ECs between 2018 and 2019 should be understood with caution as some CC and HTP dual users in 2018 might have been misclassified as CC and EC dual users. Third, due to the nature of cross-sectional national surveys, we could not assess within-person changes in tobacco use patterns over time. For example, it is unclear whether the decline in poly-tobacco use alongside increased exclusive HTP use, reflects switching among existing dual users or uptake by never users. Fourth, tobacco use was self-reported, which may be subject to reporting bias and misclassification. Fifth, participants with missing data on tobacco use were excluded from the analyses, which may have possibly introduced selection bias and affected the generalizability of our findings. Individuals who were excluded from the analytic sample tended to be female, older, and have lower incomes (Supplementary file Table 2). Last, although our analyses were descriptive with unadjusted trends, some unmeasured confounding (e.g. sexual orientation, unemployment) may still have existed^11^. For example, the dataset did not allow us to differentiate unemployed individuals and other economically inactive groups, such as housewives and students; therefore, we could not explore tobacco use trends specifically among the unemployed, a potentially vulnerable subgroup^11,17^.

Despite the limitations, this study provides robust and timely evidence on ten-year trends in tobacco use within key subgroups, reflecting the evolving tobacco landscape in Korea. A strength of this study is the assessment of within-subgroup trends over an extensive ten-year period across different types of tobacco use (CCs, ECs, HTPs, and poly-tobacco use) using nationally representative data.

## CONCLUSIONS

While overall conventional cigarette smoking has declined, persistent disparities, the clustering of tobacco use with other risk behaviors and mental illness, and the growing popularity of e-cigarette and poly-tobacco use pose new challenges in Korea. Our findings provide important insights for Korea and other countries experiencing similar challenges, highlighting the need for continued monitoring of tobacco use among vulnerable populations and for population-tailored interventions. Our findings also highlight several areas where further investigation is needed. Additional studies with larger samples of female adults are warranted to fully understand female trends of tobacco use in Korea, and longitudinal panel data are needed to elucidate individual-level transitions between tobacco products.

## Supplementary Material



## Data Availability

The data supporting this research is available from the following source: https://chs.kdca.go.kr/chs/rawDta/rawDtaProvdMain.do
