# Supplementary material for: Tobacco use trends in South Korea, 2013–2023: Persistent disparities and emerging challenges in a repeated cross-sectional study
Source: Tob Induc Dis. 2026 Feb 6;24:10.18332/tid/215655. doi: 10.18332/tid/215655 (PMC12879552; doi:10.18332/tid/215655)
Supplement: Supplementary file 1 [file TID-24-14-s1.pdf]

Appendix Table 1. Characteristics of the analytic sample (N=62,935)

	Total		2013		2023	
	n	(%)	n	(%)	n	(%)
Total	62,935	(100.0	5,792	(100.0)	5,907	(100.0)
Sex						
Male	27,434	(49.6)	2,485	(49.6)	2,574	(49.7)
Female	35,501	(50.4)	3,307	(50.4)	3,333	(50.3)
Age						
19-24	4,094	(9.4)	401	(10.2)	293	(7.5)
25-39	12,643	(25.7)	1,351	(28.0)	997	(23.8)
40-64	29,147	(47.4)	2,711	(46.5)	2,781	(47.3)
65 or older	17,051	(17.5)	1,329	(15.3)	1,836	(21.4)
Income level						
Q1 (Lowest)	15,488	(24.8)	1,430	(24.9)	1,469	(24.5)
Q2	15,702	(25.0)	1,452	(25.5)	1,467	(25.1)
Q3	15,733	(25.1)	1,430	(25.2)	1,474	(25.1)
Q4 (Highest)	15,781	(25.1)	1,434	(24.4)	1,472	(25.2)
Educational attainment						
High school or less	34,998	(56.2)	3,390	(64.1)	3,206	(52.2)
College or more	21,267	(43.8)	1,581	(35.9)	2,257	(47.8)
Occupational group						
Non-manual workers	14,441	(27.8)	1,147	(23.8)	1,385	(29.7)
Manual workers	21,381	(36.1)	1,947	(37.6)	1,956	(36.5)
Others	23,772	(36.1)	2,258	(38.6)	2,053	(33.9)
Number of health risk behaviors						
None	23676	(37.6)	2,042	(37.8)	1,706	(32.6)
One	31,816	(49.8)	2,759	(51.7)	2,935	(53.0)
Two	7,443	(12.5)	529	(10.4)	748	(14.4)
Mental illness						
Low	24,020	(68.7)	3,863	(71.7)	4,156	(70.9)
Moderate	7,844	(24.0)	1,121	(22.1)	1,182	(21.3)
Severe	2,548	(7.3)	351	(6.2)	428	(7.7)

Notes. Unweighted n, Weighted percentages. Data were from the Korea National Health and Nutrition Examination Survey, a nationally representative repeated cross-sectional survey, 2013-2023.

Appendix Table 2. Characteristics of the participants in the analytic sample and the participants excluded due to missingness on tobacco use

	Analytic sample		Excluded participants		p-value
	n	(%)	n	(%)	
Total	62,935	(100.0)	4,408	(100.0)	
Sex					
Male	27,434	(49.6)	2,184	(49.5)	$p < .001$
Female	35,501	(50.4)	2,224	(50.5)	
Age					
19-24	4,094	(9.4)	310	(7.0)	$p < .001$
25-39	12,643	(25.7)	1,010	(22.9)	
40-64	29,147	(47.4)	1,887	(42.8)	
65 or older	17,051	(17.5)	1,201	(27.3)	
Income level					
Q1 (Lowest)	15,488	(24.8)	1,291	(30.0)	$p < .001$
Q2	15,702	(25.0)	1,053	(24.5)	
Q3	15,733	(25.1)	1,036	(24.1)	
Q4 (Highest)	15,781	(25.1)	926	(21.5)	

Notes. Unweighted n, Weighted percentages. Other characteristics (i.e., education, occupational group, number of health risk behaviors, and mental illness) were omitted in this comparison because more than 95% of data on these characteristics were missing among the excluded participants.

Appendix Figure 1. Trends in the prevalence of electronic cigarettes and heated tobacco products among adult males by age (panel A), income (panel B), education (panel C), occupation (panel D), the number of health risk (panel E), and mental illness (panel F) in South Korea, 2013-2023 (N=27,434)

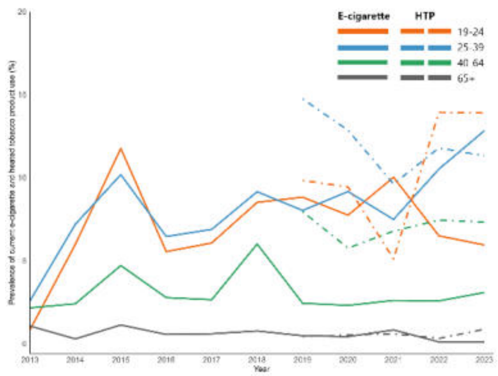

(A) Age

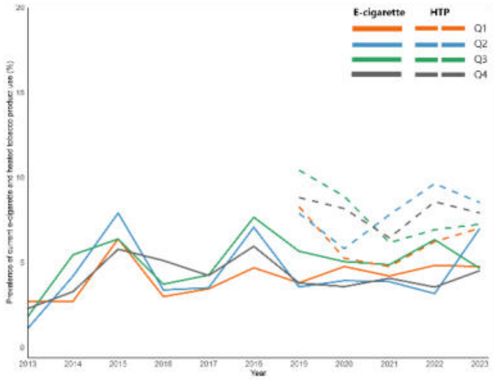

(B) Income level

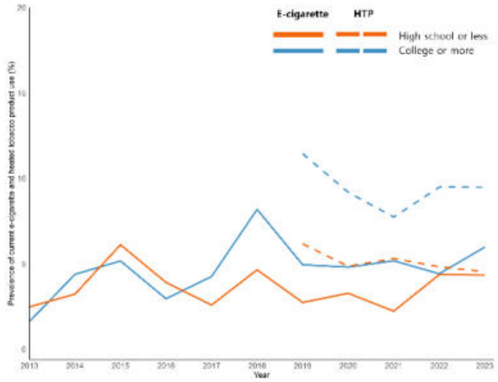

(C) Educational attainment

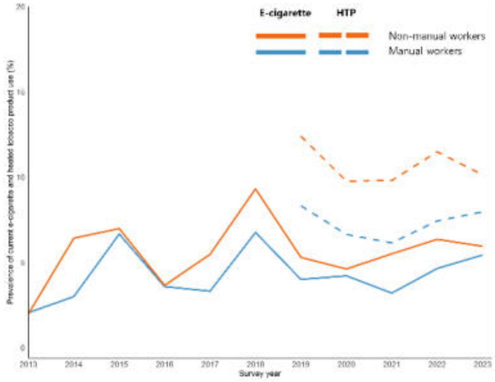

(D) Occupational groups

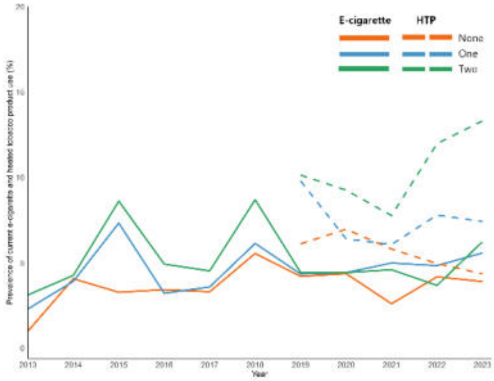

(E) Number of health risk behaviors

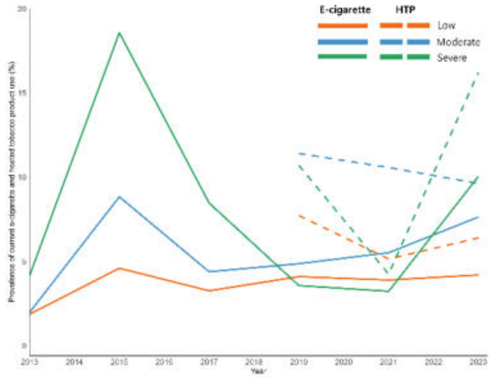

(F) Mental illness

Note: Data were from the Korea National Health and Nutrition Examination Survey, a nationally representative repeated cross-sectional survey, 2013-2023. All estimates were weighted using sampling weights provided by KNHANES.

Appendix Figure 2. Trends in poly-tobacco use among adult males by age (panel A), income (panel B), education (panel C), occupation (panel D), the number of health risk (panel E), and mental illness (panel F) in South Korea, 2013-2023 (N=27,434)

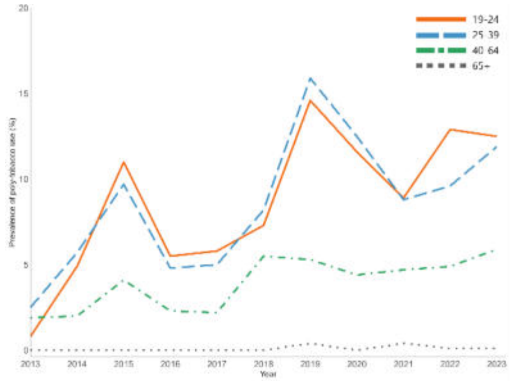

(A) Age

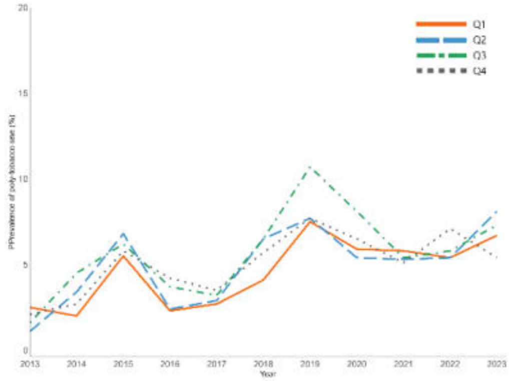

(B) Income level

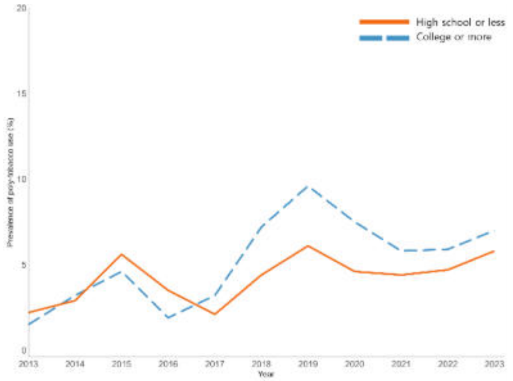

(C) Educational attainment

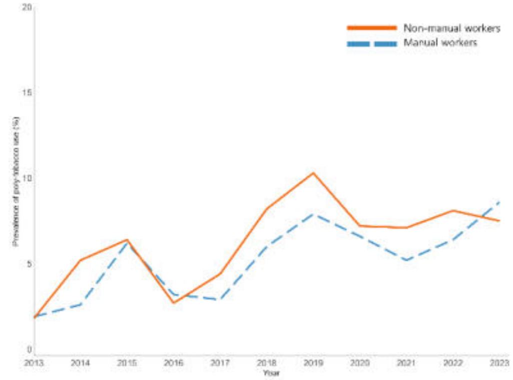

(D) Occupational groups

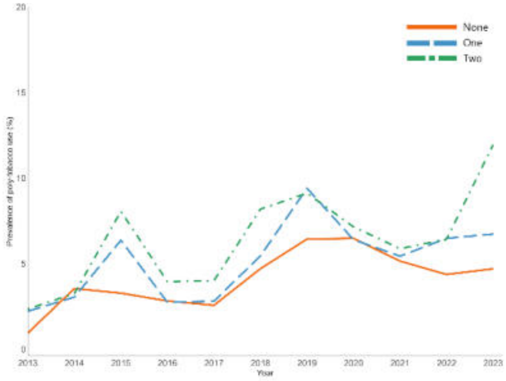

(E) Number of health risk behaviors

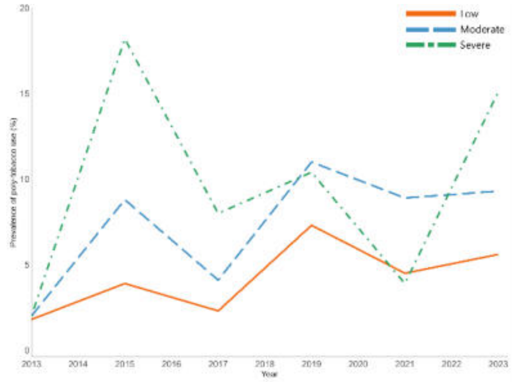

(F) Mental illness

Note: Data were from the Korea National Health and Nutrition Examination Survey, a nationally representative repeated cross-sectional survey, 2013-2023. All estimates were weighted using sampling weights provided by KNHANES.

© 2026 Lee B. et al.
